# Design of Low-Frequency Extended Signal Conditioning Circuit for Coal Mine Geophone

**DOI:** 10.3390/s25195946

**Published:** 2025-09-24

**Authors:** Zhigang Deng, Zewei Lian, Jinjiao Ye, Kai Qin, Yanbin Wang, Feng Li, Xiangfeng Meng

**Affiliations:** 1 China Coal Research Institute, Beijing 100013, China; lzw2000@alu.cau.edu.cn (Z.L.);; 2 State Key Laboratory of Coal Mine Disaster Prevention and Control, Beijing 100013, China

**Keywords:** coal mine microseismic monitoring, magnetoelectric geophone, signal conditioning circuit, low-frequency extension

## Abstract

The traditional magnetoelectric geophone is widely used in the microseismic monitoring of coal mines. However, its measurement capability in the low-frequency range is insufficient and cannot fully meet the monitoring requirements of underground coal mines, which extend as low as 0.1 Hz. This paper proposes a signal conditioning (SC) circuit based on the extended filtering method to improve the low-frequency response capability of the geophone. Through simulation and experimental tests, it is verified that the designed SC circuit can reduce the cut-off frequency of the EST-4.5C geophone from 4.5 Hz to 0.16 Hz. Meanwhile, the noise introduced by this SC circuit is relatively low thanks to its simple and easy-to-implement structural model. The test results also indicate that it provides a strong ability to resist noise interference for the geophone, which is valuable under complex working conditions. Overall, this circuit offers a feasible option for enhancing the capability of the seismic geophones used in coal mines to detect low-frequency vibration signals.

## 1. Introduction

With the in-depth mining of coal mines, dynamic disasters such as rock bursts and coal and gas outbursts have become increasingly frequent [1]. Microseismic events are often precursors of these disasters [2]. At present, microseismic monitoring systems are widely deployed in underground coal mines [3]. By capturing the elastic vibration wave signals released by the fracture of coal and rock masses, functions such as source location [4], energy calculation [5], and stress distribution inversion [6] can be achieved. According to research, vibration wave signals will attenuate when propagating in coal strata, and the higher the main frequency of the signal, the faster the attenuation [7]. This leads to the fact that high-energy vibration waves exhibit a relatively low main frequency when propagating over long distances [8]. Therefore, the microseismic monitoring system requires a wide frequency band monitoring range and sufficient capability to detect low-frequency vibration signals (below 1 Hz, even as low as 0.1 Hz) [9].

The microseismic monitoring system first receives vibration signals through sensors [10]. Currently, the sensors most commonly used in microseismic monitoring systems deployed in underground coal mines are primarily seismometers and magnetoelectric geophones [11]. Seismometers perform well in picking up low-frequency vibration signals (especially below 1 Hz) [12,13]. But their monitoring range is usually 0.01–50 Hz, and they are large in size and weight, with high cost and power consumption [14]. Therefore, their wide application in underground coal mines still has limitations. The magnetoelectric geophone is smaller in size and weight, has lower cost and power consumption, and exhibits a strong ability to pick up medium- and high-frequency signals (typically ranging from a few Hz to several hundred Hz). It is suitable for monitoring most microseismic events [15]. However, traditional geophones have a relatively weak ability to pick up low-frequency vibration signals [16]. To meet the low-frequency requirements of microseismic monitoring in coal mines, it is necessary to optimize the geophone performance.

Researchers have conducted extensive work on the low-frequency extension of magnetoelectric geophones. Yao et al. [17] optimized the material and structure of the spring sheet to expand the seismograph’s bandwidth. Barzilai et al. [18] used capacitive feedback to extend the seismograph’s frequency range. Collette et al. [19] proposed a sensor fusion method. With the development of information technology, analog and digital technologies have been fully utilized. Kinoshita [20] designed a filtering algorithm to achieve low-frequency compensation of the discrete inverse response. Zhang et al. [21] implemented feedback regulation through a PID controller and reduced the natural frequency of the seismograph to 0.166 Hz. Digital technology does not rely on precise parameters of electronic components and is highly resistant to interference. However, its frequency expansion effect is limited by the computer’s processing power, which cannot fully support the real-time, uninterrupted monitoring requirements of microseismic activity in coal mines. Analog technology has been proven to have excellent frequency extension capabilities and real-time performance [22]. Currently, the widely studied analog technology primarily compensates for the transfer function by utilizing series correction circuits at the output end of a single geophone. Choi et al. [23] designed a state variable filter (SVF), which effectively suppresses the formant of the seismic geophone and extends the cut-off frequency to 0.8 Hz. Yang et al. [24] implemented the integration of seismometers based on the geophone bandwidth extension (BE) circuit, which extends the cut-off frequency of short-period seismometers from 4.5 Hz to 0.2 Hz. Ma et al. [25] ingeniously combined the concepts of negative resistance and correction circuits, reduced the cut-off frequency of the geophone from 10 Hz to 0.7 Hz, and fully leveraged the advantages of analog technology, achieving remarkable results.

This paper considers the application requirements of the microseismic monitoring systems in underground coal mines and proposes a signal conditioning (SC) circuit for magnetoelectric geophones based on the principle of extended filtering. This circuit model is simple and easy to implement, and the electronic components used are highly economical and readily available. This research also considers the noise environment in underground coal mines and the self-noise of circuits, and it incorporates links that mitigate the impact of noise. We obtain data through simulation and experiments and compare them with those of the original geophone. The results show that the proposed signal conditioning circuit can extend the cut-off frequency of the magnetoelectric geophone from 4.5 Hz to 0.16 Hz. Overall, this design assists in monitoring low-frequency vibration signals in underground coal mines.

## 2. Principle and Model of the Geophone

### 2.1. Working Principle of the Geophone

**Figure 1:**
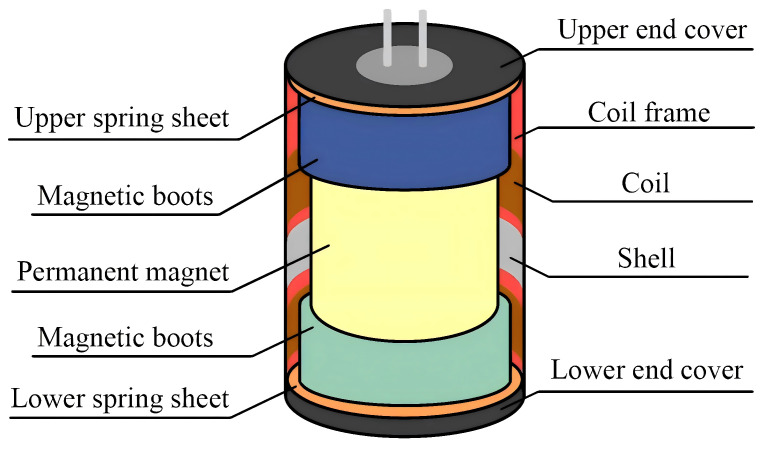
Geophone structure.

The internal structure of the magnetoelectric geophone is shown in Figure 1. The geophone housing is a cylindrical soft iron, and the interior is a magnetic circuit structure, including a permanent magnet and a magnetic shoe. The soft iron casing and magnetic boots seal the magnetic circuit inside the casing. The permanent magnet is fixedly connected to the casing. The function of the magnetic boots is to make the internal magnetic field more uniform. The vibration system part is located between the permanent magnet and the inner wall of the sensor housing. It is an inertial system composed of coils, coil frames, and springs.

**Figure 2:**
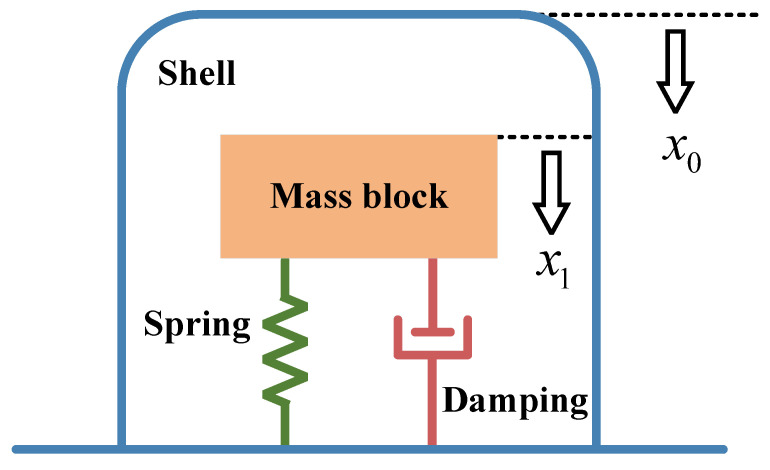
Geophone dynamic model.

The working principle of the magnetoelectric geophone is based on Faraday’s law of electromagnetic induction. Its dynamic model can be simplified as a single-degree-of-freedom second-order system composed of a mass block, damping, and spring, as shown in Figure 2.

When the ground vibrates, the geophone housing and the internal system sense the vibration. Due to the presence of the spring, the internal mass block moves relatively to the housing and the permanent magnet. At this time, the displacement of the housing and the permanent magnet is denoted as $x_{0}$, and the displacement of the mass block is denoted as $x_{1}$. Then, the induced electromotive force generated by the coil cutting the magnetic field lines is

(1) $$U=NBlv=NBl\left({\dot{x}}_{1}-{\dot{x}}_{0}\right)$$

where *N* represents the number of coil turns; *B* represents the magnetic induction intensity; *l* represents the effective length of a single-turn coil; and *v* represents the relative velocity between the mass body and the casing, as well as the permanent magnet.

The kinematic equation of the geophone is

(2) $$m{\ddot{x}}_{1}+c\left({\dot{x}}_{1}-{\dot{x}}_{0}\right)+k\left(x_{1}-x_{0}\right)=0$$

where *m* represents the mass of the mass body, *c* represents the damping coefficient, and *k* represents the spring stiffness coefficient. Let $x=x_{1}-x_{0}$, and perform Laplace transforms on Formulas (1) and (2) to obtain, respectively,

(3) $$U_{0}\left(s\right)=NBlsX\left(s\right)$$

(4) $$\left(ms^{2}+cs+k\right)X\left(s\right)+ms^{2}X_{0}\left(s\right)=0$$

The transformation of Formula (4) yields

(5) $$H_{0}\left(s\right)=\frac{X(s)}{X_{0}\left(s\right)}=\frac{-ms^{2}}{ms^{2}+cs+k}$$

Due to the relationship between the working characteristics and structural parameters of the geophone, its natural angular frequency $\omega_{0}=\sqrt{k/m}$, damping ratio $\xi_{0}=c/2\sqrt{km}$, and sensitivity K=NBl; then, Formula (3) can be expressed as

(6) $$U_{0}\left(s\right)=NBlsH_{0}\left(s\right)X_{0}\left(s\right)=\frac{-Ks^{2}}{s^{2}+2\xi_{0}\omega_{0}s+\omega_{0}^{2}}sX_{0}\left(s\right)$$

Then, the input–output transfer function of the system is

(7) $$H_{0}\left(s\right)=\frac{-Ks^{2}}{s^{2}+2\xi_{0}\omega_{0}s+\omega_{0}^{2}}$$

**Figure 3:**
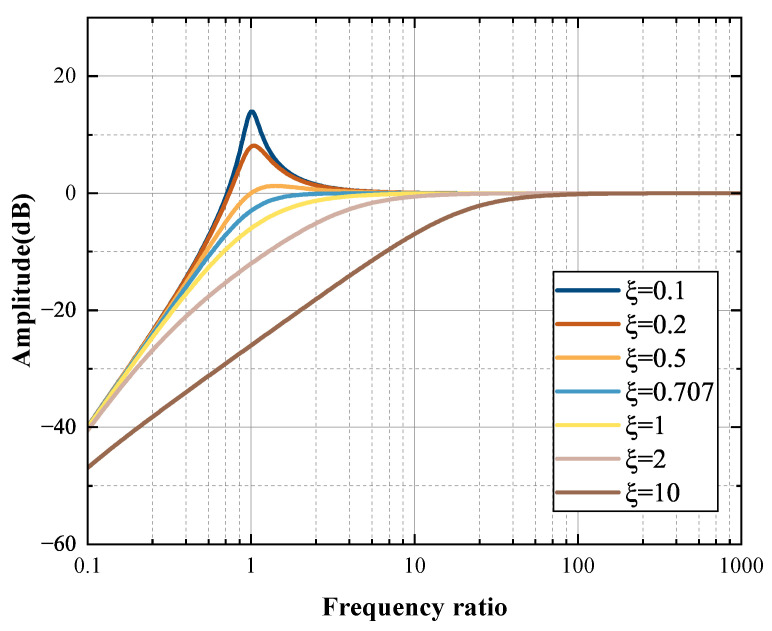
The amplitude–frequency characteristic curves of geophones with different damping ratios.

The ratio of the frequency to be measured to the natural frequency of the geophone is taken as the X-axis; the amplitude is taken as the Y-axis; and the damping ratios are 0.1, 0.2, 0.5, 0.707, 1, 2, and 10. After normalization, the amplitude–frequency characteristics of the geophone are as shown in Figure 3.

According to Formula (7) and Figure 3, it can be known that the amplitude–frequency characteristics of the magnetoelectric geophone are characterized as a second-order high-pass filter. The geophone has a significant attenuation effect on signals below its cut-off frequency, and the lower damping ratio will also cause a resonance peak in the amplitude response of the geophone at its natural frequency. Therefore, in order to enable the geophone to have a stable low-frequency vibration signal capability, it is necessary to adjust its cut-off frequency and damping ratio reasonably.

### 2.2. Equivalent Electrical Model of the Geophone

In order to facilitate the observation of the changes in the input and output characteristics of the geophone before and after adding the SC circuit, as well as to adjust the capacitance and resistance values of the circuit reasonably, this article obtains an equivalent electrical model of the geophone based on the mechanical–electrical analogy principle [26], as shown in Table 1.

Among Table 1, *c* represents the damping coefficient of the geophone, and *R*, *L*, and *C* are the resistance, coil inductance, and capacitance in the equivalent electrical model circuit of the geophone, respectively. This paper selects the EST-4.5C geophone produced by Baoding Langte Company as the research object. According to the technical parameters provided by the manufacturer, substituting them into Table 1 yields Table 2.

**Figure 4:**
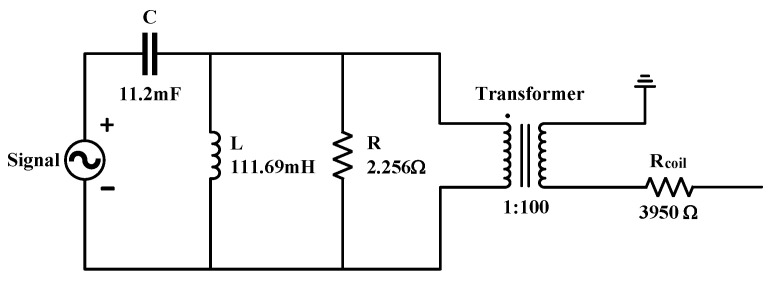
Electrical equivalent model diagram of the magnetoelectric geophone.

Based on the above data, the electrical equivalent model of the magnetoelectric geophone can be obtained as shown in Figure 4, where $R_{\mathrm{coil}}$ is the internal resistance of the geophone coil.

**Figure 5:**
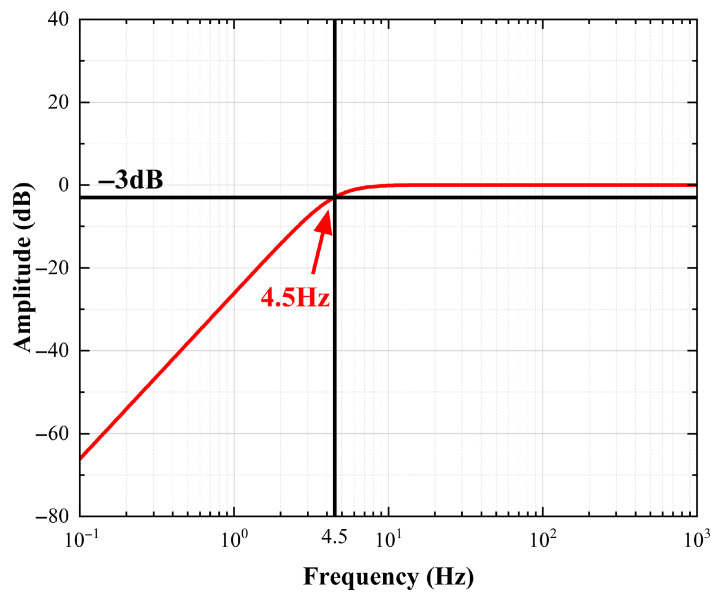
The amplitude–frequency characteristic curve of the EST-4.5C geophone.

The frequency response is tested using the simulation software LTspice 24. The frequency range is set to 0.1–1000 Hz, and the amplitude–frequency characteristic curve of the model is obtained. After normalization processing, the results are as shown in Figure 5.

Combining the curves in Figure 3 with those in Figure 5, it can be known that this electrical model can fully restore the amplitude–frequency characteristic of the geophone. This model lays the foundation for designing the geophone signal conditioning circuit, adjusting its parameters, and analyzing the input and output characteristics, which is conducive to improving the accuracy of the subsequent model.

## 3. Method and Design of Signal Conditioning Circuit

### 3.1. Analysis of the Principle of Extended Filtering Method

**Figure 6:**
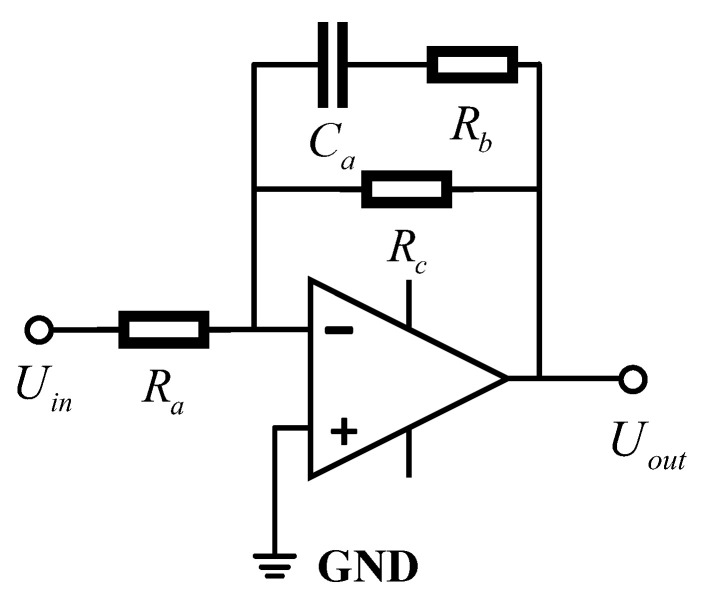
Extended filter circuit structure diagram.

This paper considers the demand for low-frequency vibration signal monitoring in underground coal mines and proposes a signal conditioning circuit for a magnetoelectric geophone. The circuit is implemented based on the extended filtering method. A correction circuit is connected in series at the output end of the geophone to improve its ability to pick up low-frequency vibration signals. The design of the extended filter circuit is aimed at improving the response to low-frequency signals. It does not rely on zero-pole matching between the transfer function of the geophone and the correction circuit. Therefore, the requirements for the parameter accuracy of the electronic components used are not very strict. The structure of the extended filter circuit is shown in Figure 6.

The transfer function of the extended filter circuit is

(8) $$H_{1}\left(s\right)=\frac{U_{\mathrm{out}}\left(s\right)}{U_{\mathrm{in}}\left(s\right)}=\frac{R_{c}\left(R_{b}C_{a}s+1\right)}{R_{a}\left[C_{a}\left(R_{b}+R_{c}\right)s+1\right]}$$

where the gain of the circuit is $K_{1}=R_{c}/R_{a}$, the time parameter is $\tau_{1}=C_{a}\left(R_{b}+R_{c}\right)$, and $\tau_{2}=C_{a}R_{b}$.

Since the transfer function of the magnetoelectric geophone is characterized as a second-order high-pass filter, in order to achieve more effective frequency extension, this paper selects two extended filter circuits with the same parameters. They form a second-order extended filter circuit through a cascade method. Based on the relationship between the time parameter and the cut-off angular frequency $\omega_{1}=1/\tau_{1},\omega_{2}=1/\tau_{2}$, the transfer function can be obtained as follows:

(9) $$H_{2}\left(s\right)=\frac{K_{1}^{2}\omega_{1}^{2}\left(s^{2}+2\omega_{2}s+\omega_{2}^{2}\right)}{\omega_{2}^{2}\left(s^{2}+2\omega_{1}s+\omega_{1}^{2}\right)}$$

Combining Formulas (7) and (9), the transfer function of the geophone after the series extension filter circuit is obtained as

(10) $$H_{3}\left(s\right)=\frac{-Ks^{2}}{s^{2}+2\xi_{0}\omega_{0}s+\omega_{0}^{2}}\cdot \frac{K_{1}^{2}\omega_{1}^{2}\left(s^{2}+2\omega_{2}s+\omega_{2}^{2}\right)}{\omega_{2}^{2}\left(s^{2}+2\omega_{1}s+\omega_{1}^{2}\right)}$$

To obtain the cut-off frequency of this transfer function, Fourier transform needs to be performed on Formula (10). The transformation result is shown in Formula (11):

(11) $$H_{3}\left(j\omega \right)=\frac{K\omega^{2}}{-\omega^{2}+2j\xi_{0}\omega_{0}\omega +\omega_{0}^{2}}\cdot \frac{K_{1}^{2}\omega_{1}^{2}\left(-\omega^{2}+2\omega_{2}\omega j+\omega_{2}^{2}\right)}{\omega_{2}^{2}\left(-\omega^{2}+2\omega_{1}\omega j+\omega_{1}^{2}\right)}$$

Perform the modulus operation on Formula (11) and, based on the definition of the cut-off frequency of the transfer function

(12) $$20\log\frac{|H_{3}\left(j\omega \right)|}{KK_{1}^{2}\left(\omega_{1}^{2}/\omega_{2}^{2}\right)}=-3dB$$

The cut-off frequency of the new geophone system after extended filtering can be obtained to satisfy the following relationship:

(13) $$\frac{2\omega^{4}{\left(\omega^{2}+\omega_{2}^{2}\right)}^{2}}{{\left(\omega^{2}+\omega_{1}^{2}\right)}^{2}}={\left(\omega_{0}^{2}-\omega^{2}\right)}^{2}+4\xi_{0}^{2}\omega_{0}^{2}\omega^{2}$$

It can be known from Formula (13) that the cut-off angular frequency of the new system ω is related to $\omega_{0}$, $\omega_{1}$, $\omega_{2}$, $\xi_{0}$. In order to better perform parameter matching calculation, let $\omega_{0}=\omega_{2}$, $\xi_{0}=1$; then, Formula (13) can be simplified as

(14) $$\sqrt{2}\omega^{2}=\omega^{2}+\omega_{1}^{2}$$

Finally, Formula (14) can be obtained as follows:

(15) $$\omega =\frac{1}{\sqrt{\sqrt{2}-1}}\omega_{1}$$

Through the above analysis, the relationship expressions among the cut-off frequency of the magnetoelectric geophone, the cut-off frequency of the new system after extended filtering, and the cut-off frequency of the extended filtering circuit were obtained. Combined with the target frequency range, the parameters of the electronic components in the circuit can be inferred backward.

**Figure 7:**
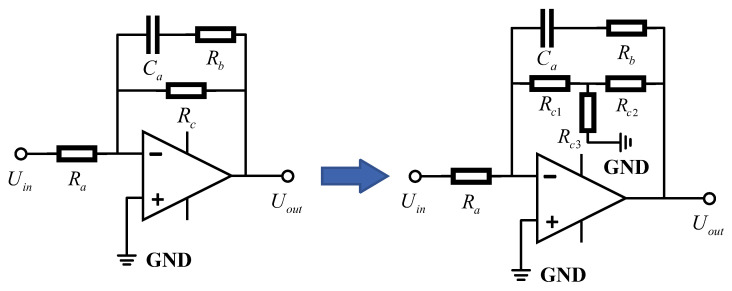
The transformed extended filter circuit structure diagram.

During the process of parameter design, we found that a low cut-off frequency would result in a larger value of the resistance $R_{c}$. However, large resistors in circuits are often accompanied by high noise and are more susceptible to temperature drift. To reduce its impact, we used an asymmetric T-type resistor network instead of a large resistor [27,28]. The variation in the extended filter circuit is shown in Figure 7.

The resistance relationship before and after transformation is satisfied:

(16) $$R_{c}=R_{c1}+R_{c2}+\frac{R_{c1}R_{c2}}{R_{c3}}$$

Under the condition that $R_{c1}+R_{c2}+R_{c3}<R_{c}$, by selecting reasonable parameters, the T-type resistor network can replace a single resistor. This approach not only retains the frequency expansion effect of the circuit but also reduces the negative impact caused by large resistors.

### 3.2. Comprehensive Design of Signal Conditioning Circuits

**Figure 8:**
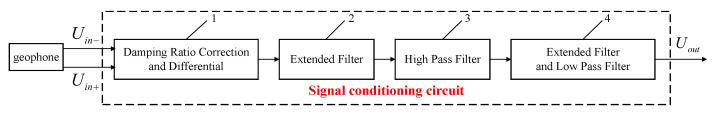
Flowchart of the signal conditioning circuit.

The signal conditioning circuit of the magnetoelectric geophone proposed in this paper includes four links: damping ratio correction and differential, extended filter, high-pass filter, and low-pass filter. The overall process is shown in Figure 8.

In Section 3.1, we analyzed the principle of the extended filtering method and set the damping ratio to $\xi_{0}=1$. Therefore, in the first section of Figure 8, the initial damping ratio was adjusted by paralleling a resistor at the output end of the geophone [26]. The implementation principle is as follows:

(17) $$\xi_{0}=\frac{c}{2m\omega_{0}}+\frac{K^{2}}{\left(R_{\mathrm{coil}}+R_{\mathrm{par}}\right)2m\omega_{0}}=1$$

where $R_{\mathrm{par}}$ is the resistance value of the parallel resistor. Based on the parameters of EST-4.5C and the design template, $R_{\mathrm{par}}=48.68\;$kΩ in this paper.

Due to the complex working conditions and environmental background in underground coal mines, the microseismic data picked up are accompanied by a large amount of noise interference [29]. Differential circuits are considered to perform well in noise suppression [30]. In the first section of Figure 8, an operational amplifier group is applied to form a differential circuit to improve the signal-to-noise ratio of the picked signal.

**Figure 9:**
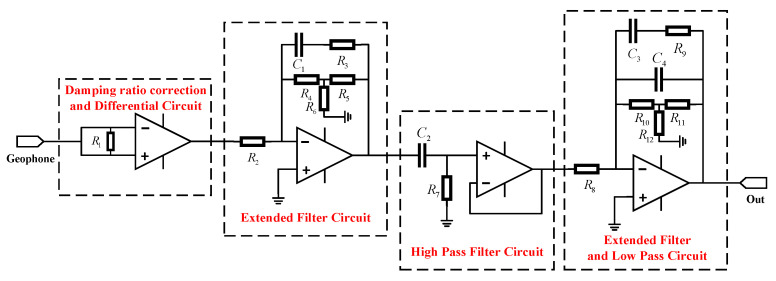
Signal conditioning circuit structure diagram.

In the signal conditioning circuit, active high-pass filter circuits and low-pass filter circuits are added in the third and fourth sections of Figure 8, respectively, to reduce the influence of DC bias, zero drift, and high-frequency interference. The cut-off frequency settings of the two types of filters should ensure a flat passband within the target frequency range. Therefore, the cut-off frequencies of the two filters selected in this paper are 0.05 Hz and 150 Hz, respectively. To simplify the circuit structure and reduce the number of operational amplifiers, thereby minimizing interferences such as power consumption and noise, a capacitor is connected in parallel at the feedback terminal of the operational amplifier in the second extended filter circuit. This enables the circuit to exhibit both extended filtering and low-pass filtering characteristics simultaneously. The complete signal conditioning circuit of the magnetoelectric geophone is shown in Figure 9.

When signal conditioning is carried out using a series correction circuit, the electronic components in the circuit introduce electronic noise, which affects the low-frequency expansion effect. These noises typically originate from resistive thermal noise, as well as equivalent voltage and current source noise at the input end of operational amplifiers [31,32,33]. To reduce the influence of electronic noise, operational amplifiers with better performance are usually selected in the early stages of circuit design, and the resistance value is reduced as much as possible [34,35,36,37]. In the analysis in Section 3.1, we learned that the cut-off frequency of the extended filter circuit is inversely proportional to both the capacitance $C_{a}$ and resistance $R_{b}$, $R_{c}$. Furthermore, when the cut-off frequency is determined, the capacitance value is also inversely proportional to the resistance value. This indicates that, when the cut-off frequency is set relatively low, the capacitance and resistance values in the circuit will be correspondingly larger. If the capacitance value is too large, it will affect the circuit’s response speed; if it is too small, it will easily cause signal distortion and, at the same time, increase the resistance value, introducing more intense noise interference. Based on the above analysis, when selecting parameters, we uniformly set the capacitance value to 10 μF. Meanwhile, we replaced the resistors in each extended filter circuit with an asymmetric T-shaped equivalent resistance network. Through mathematical calculations, we selected the appropriate coefficients to reduce the noise introduced by this equivalent resistance network without changing the cut-off frequency of the circuit. In this paper, $R_{4}=R_{5}=R_{10}=R_{11}=1/6R_{c}$, $R_{6}=R_{12}=1/24R_{c}$. Selecting the coefficient in this way can minimize the resistance value error between the theoretical analysis and the actual design in this manuscript to the greatest extent. 

### 3.3. Simulation Verification of Signal Conditioning Circuits

**Figure 10:**
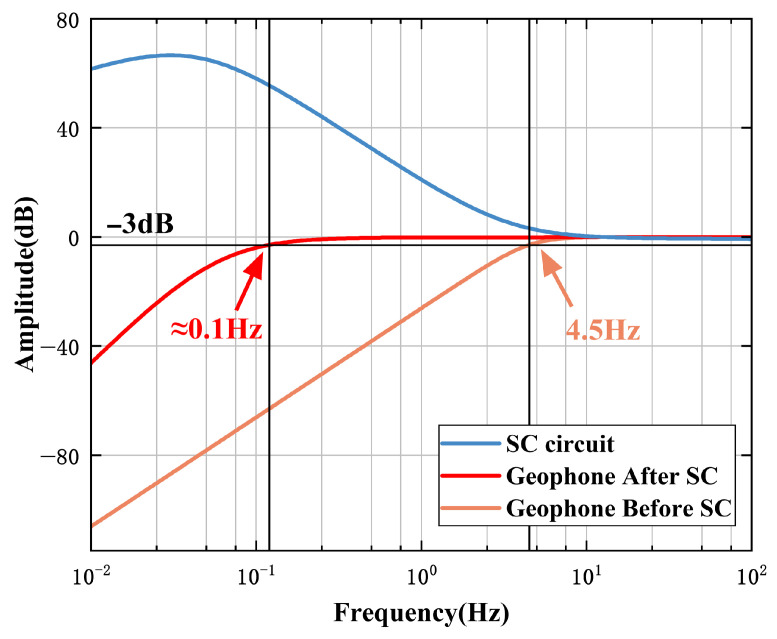
The amplitude–frequency characteristic diagrams of the SC circuit and the seismic geophone before and after SC.

In order to preliminarily verify the feasibility of the signal conditioning circuit of the designed magnetoelectric geophone, we simulated the amplitude–frequency characteristics of the signal conditioning circuit and the geophone before and after signal conditioning, respectively, as shown in Figure 10.

Given the characteristic that the gain of the geophone rapidly attenuates in the frequency band below 4.5 Hz, the signal conditioning circuit provides full compensation. It maintains zero gain in the frequency band above 4.5 Hz. Eventually, the cut-off frequency of the geophone after signal conditioning is reduced from 4.5 Hz to around 0.1 Hz while maintaining a flat passband with zero gain, and the attenuation of the stopband is also relatively smooth. The simulation results preliminarily verify that the signal conditioning circuit designed based on the extended filtering method has a low-frequency extension effect on the geophone, which aligns with the theoretical analysis.

## 4. Results and Analysis of Signal Conditioning Circuit

### 4.1. Hardware Implementation of Signal Conditioning Circuit

**Figure 11:**
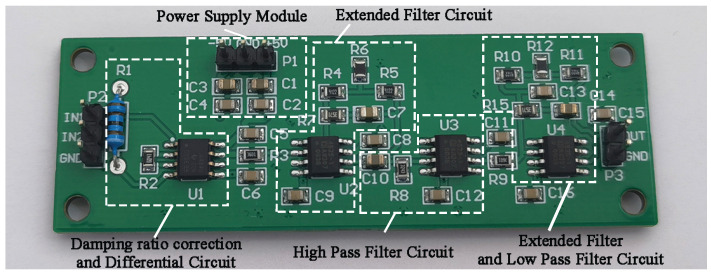
Signal conditioning circuit board diagram.

The designed signal conditioning circuit board is shown in Figure 11, which includes each filter bank, input, output, and power supply module. The circuit is primarily composed of operational amplifiers, basic resistors, and capacitors, ensuring easy component availability, convenient electrical design, and economic benefits.

Considering that operational amplifiers can introduce electronic noise, in this paper, based on the performance parameters of operational amplifiers, the AD8422 instrumentation amplifier (Norwood, MA, USA) is selected in the differential circuit to control the gain through a single resistor. The multi-stage filter circuit selected the ADA4528 operational amplifier (Norwood, MA, USA). These two types of operational amplifiers have the advantages of a low offset voltage and current, low bias current, low voltage and current noise density, high gain-bandwidth product, high voltage slew rate, sufficient open-loop gain, and high common-mode rejection ratio. At the same time, they are manufactured by Analog Devices (ADI), thus being highly economical and readily available. These characteristics provide strong support for the application of signal conditioning circuits.

### 4.2. Test Results and Analysis of the Vibration Table

**Figure 12:**
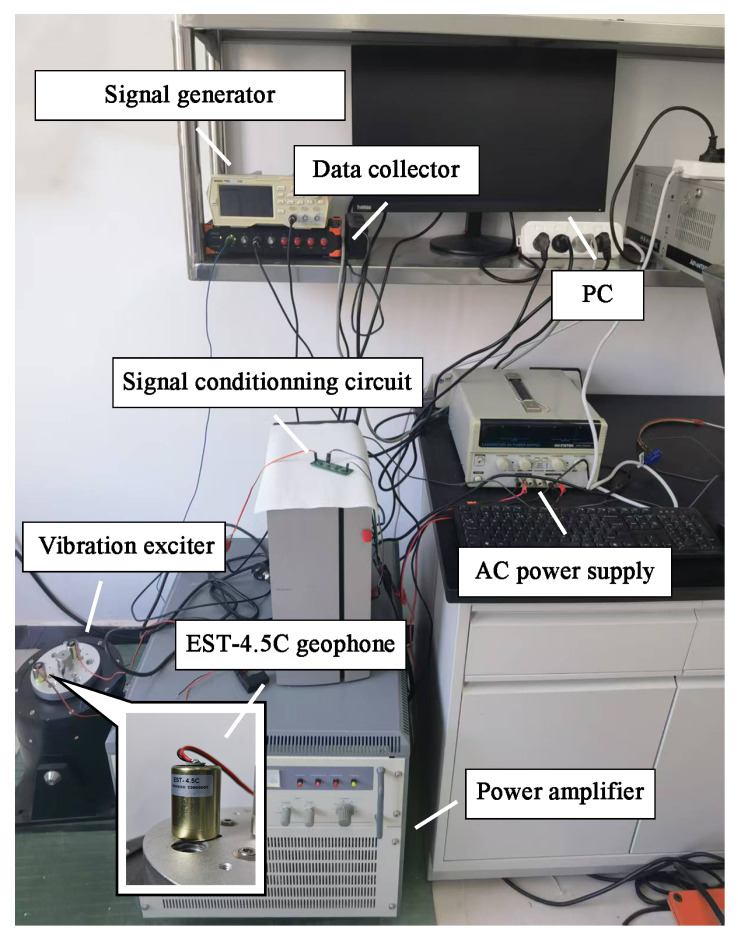
Vibration table system.

To verify the true effect of the signal conditioning circuit, we tested the frequency response curve of the geophone when connected to the conditioning circuit via the vibration table system (KingSci Instruments, Beijing, China). Its overall structure is shown in Figure 12. The signal generator emits sinusoidal vibration signals with variable frequencies and amplitudes. The power amplifier amplifies these signals before being connected to the exciter. The exciter generates the corresponding vibration excitation, which is then converted by the geophone into a voltage signal for output. This electrical output signal is sent to the data acquisition instrument after passing through the signal conditioning circuit. The collected data can be displayed and analyzed on the PC.

**Figure 13:**
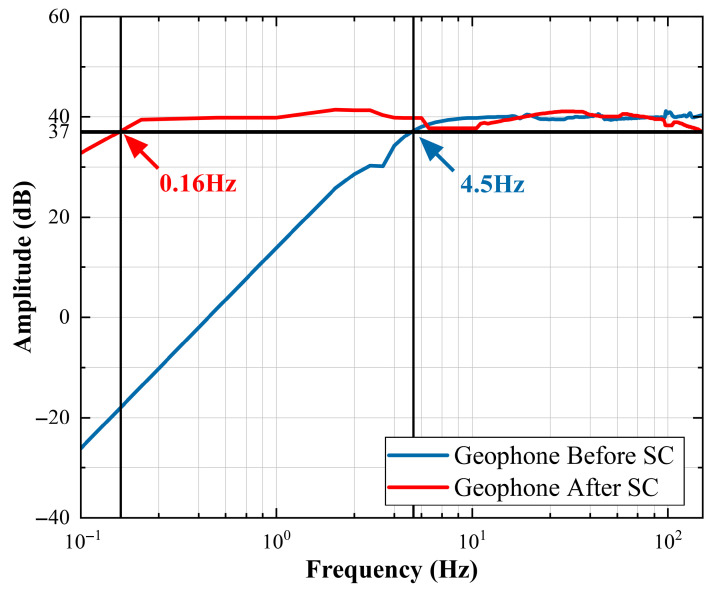
The amplitude–frequency characteristic curve graph of the geophone measured in the experiment.

For the test experiment, we placed a geophone, which was connected directly to the data acquisition instrument, on the exciter. The experiment utilized the AC sweep frequency function of the signal generator to emit signals at various frequencies continuously. The data collected on the PC were collated, and the amplitude–frequency characteristic curves of the EST-4.5C magnetoelectric geophone before and after the series signal conditioning circuit were obtained, as shown in Figure 13.

As shown in Figure 12, the cut-off frequency of the geophone before signal conditioning is 4.5 Hz. After signal conditioning, this drops to 0.16 Hz. The gain of the source signal within the passband is consistently around 40 dB, which is consistent with the geophone’s initial sensitivity of 100 V/(m/s). These results indicate that the signal conditioning circuit effectively enhances the magnetoelectric geophone’s ability to detect low-frequency vibrations. Additionally, the geophone’s gain slightly decreases within the 4.5–10 Hz range after signal conditioning. This is because, after the damping ratio adjustment, the cut-off frequency of the geophone is higher than 4.5 Hz, and the parameters of the actual circuit components cannot be completely consistent with theory. This issue can be resolved through fine-tuning the resistance element.

**Figure 14:**
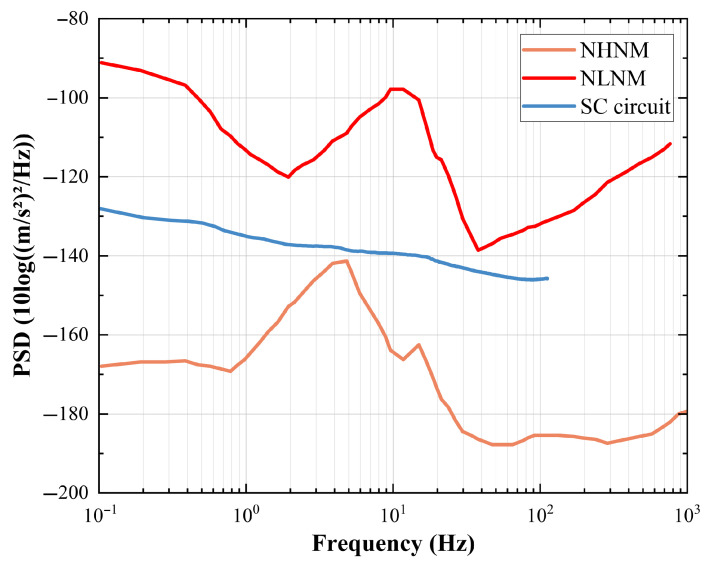
Comparison chart of signal conditioning circuit and the new model PSD of Earth background noise.

When evaluating the performance of the signal conditioning circuit, the background noise generated by the circuit itself must also be considered. Due to the attenuation of the microseismic signal after propagation through coal and rock strata, the signal is weak by the time it reaches the geophone. Circuit noise is amplified alongside the picked signal during the conditioning process. If the circuit noise is significant, the effective signal will be obscured, potentially leading to microseismic events being misjudged or missed. Therefore, in addition to the amplitude–frequency characteristic curve, the noise level is also an important indicator when evaluating the performance of signal conditioning circuits. This process involves comparing the power spectral density (PSD) of the circuit noise with that of the new Earth background noise model [38]. We tested the signal conditioning circuit separately on the vibration table system (KingSci Instruments, Beijing, China). We obtained the PSD of the self-noise of the signal conditioning circuit through spectral analysis and calculation. The results of the comparison with the new model of Earth background noise are shown in Figure 14.

NHNM and NLNM represent the new high Earth noise model and the new low Earth noise model, respectively. The noise power spectral density of the tested signal conditioning circuit is generally found within the envelope of NHNM and NLNM at frequencies above 0.1 Hz. Therefore, it can be considered that the noise level of the signal conditioning circuit is qualified. Combined with the test results of the amplitude–frequency characteristics, it can be demonstrated that the geophone, after signal conditioning, can pick up low-frequency vibration signals.

**Figure 15:**
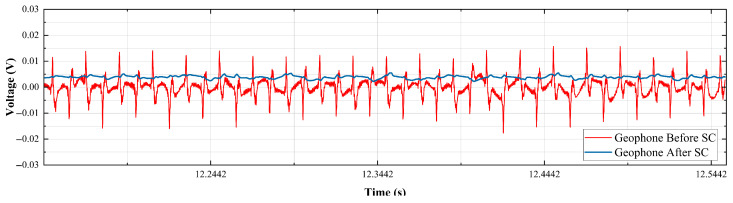
Geophone noise test waveform diagram.

Furthermore, in order to verify that the designed signal conditioning circuit can resist the interference of external power frequency noise and improve the ability of the geophone to pick up effective signals in the complex environment of underground coal mines, we simulate a noise environment in the laboratory for testing. The equipment used for the test has low energy but obvious noise. The device is placed close to the geophone to start it. The collected data are displayed on the PC end. One of the clips is shown in Figure 15.

The test snippet reveals that the signal waveform captured by the original geophone in the noisy environment exhibits severe glitches, with a period of 0.02 s, which aligns with the frequency characteristics of power frequency noise (50 Hz). The waveform glitch picked up by the geophone after signal conditioning is improved, and the overall amplitude is decreased. This indicates that the signal conditioning circuit plays a certain role in suppressing power frequency noise.

## 5. Conclusions

The magnetoelectric geophone, as the most commonly used vibration sensor in the microseismic monitoring system of underground coal mines, restricts the performance of the microseismic monitoring system due to its working characteristics. Due to its strong attenuation characteristics for signals lower than its cut-off frequency, the traditional geophone can no longer meet the monitoring requirements of low-frequency vibration signals in the coal mine microseismic monitoring system. This paper, examining the characteristics of the geophone and considering the underground environment of coal mines, proposes a signal conditioning circuit for the low-frequency expansion of coal mine seismic geophones. The circuit design is based on the principle of the extended filtering method and incorporates damping ratio correction and differential, as well as high-pass and low-pass filtering links, to assist in adjusting the circuit properties.

This paper selected the EST-4.5C geophone with high sensitivity and an appropriate damping ratio as the research object. Based on the data of this geophone, we accurately restored its electrical model in simulation software. Starting from this model, the structure and parameters of the signal conditioning circuit were designed. The SC circuit reduced the use of operational amplifier groups and large numerical resistors through structural optimization. An experiment was conducted using vabration table system. The data show that the signal conditioning circuit could reduce the cut-off frequency of the EST-4.5C magnetoelectric geophone from 4.5 Hz to 0.16 Hz. We also tested the self-noise level and the ability to resist the external interference of the signal conditioning circuit in a quiet environment and a simulated power frequency noise environment. The results show that the noise level of the circuit meets the qualification. Moreover, this circuit has a certain ability to suppress external noise interference.

Combining theoretical analysis and experimental tests, this paper demonstrates that the signal conditioning circuit enhances the magnetoelectric geophone’s ability to detect low-frequency vibration signals. Meanwhile, the circuit design is simple and easy to implement, providing strong support for the need to fully arrange geophones in underground coal mines to pick up low-frequency vibration signals.

## Figures and Tables

**Table 1:** Equivalent electrical model of the geophone.

Mechanical System	Electrical System
$F=m\frac{dv}{dt}\;$	$i=C\frac{du}{dt}$
F=k∫vdt	$i=\frac{1}{L}\int u\;dt$
F=cv	i=u/R
$\omega_{0}^{2}=k/m$ $\;\;2\xi_{0}\omega_{0}=c/m$	$\omega_{0}^{2}=1/LC$ $\;\;\;2\xi_{0}\omega_{0}=1/RC$

**Table 2:** Equivalent circuit parameters of the EST-4.5C geophone.

Component Parameters	Numerical Value
C=m	0.0112 (F)
L=1/k	111.69 (mH)
R=1/c	2.256 (Ω)
Transformer Turns Ratio 1:*K*	1:100

## Data Availability

Data are contained within the article.
